# Resection of Contralateral Scapular Oligometastasis in Non-small Cell Lung Cancer Post Right Salvage Pneumonectomy

**DOI:** 10.7759/cureus.39790

**Published:** 2023-05-31

**Authors:** Adam Djouani, Teja Maddipati, Alexander Smith, Lawrence Okiror

**Affiliations:** 1 Thoracic Surgery, Guy's and St Thomas' NHS Foundation Trust, London, GBR

**Keywords:** thoracic oncosurgery, salvage procedure, post pneumonectomy, non-small cell lung carcinoma (nsclc), oligometastatic

## Abstract

A 74-year-old male was diagnosed with right hilar T4N1M0 squamous cell carcinoma of the lung. Radical oncological treatment was initiated with curative intent. Despite this, a post-operative computed tomography scan showed residual disease. Therefore, right thoracotomy and salvage pneumonectomy were performed. The patient recovered well post-operatively. Unfortunately, seven months later, he re-presented with a left scapula subcutaneous mass, with a biopsy confirming metastatic lung squamous cell carcinoma. Radiotherapy was not possible as it would have irradiated the remaining lung, and therefore, surgical resection and chest wall reconstruction were undertaken. The patient remains free of disease at 6 months follow-up. We present an interesting case of surgical management of oligometastatic lung cancer.

## Introduction

Lung cancer remains the leading cause of cancer-related deaths in the UK [1]. A total of 85% of lung cancer cases are non-small cell lung cancer (NSCLC) which comprises adenocarcinoma, squamous cell carcinoma, and large cell carcinoma [2]. Cigarette smoking remains the most important risk factor. Curative surgical management for NSCLC is offered to medically fit patients with early-stage disease. In contrast, the prognosis for patients with advanced non-small cell lung cancer (NSCLC) has historically been poor. However, with developments in chemotherapy and novel immunotherapeutic approaches, outcomes are improving. Despite this, failure of treatment continues to occur with several acquired mutations known to render tumors treatment-resistant [3]. Salvage surgery may be defined as the surgical resection of disease following the failure of other treatment modalities where surgical resection was not the primary intended mode of treatment [4]. In the context of NSCLC cancer, salvage surgery is an option in individuals where chemo-radiotherapy/immunotherapy has down-staged unresectable disease to resectable, and in cases where initial oncological therapy has failed or where disease relapse has occurred following an initial treatment response. Surgical management of disease recurrence in NSCLC has historically been confined to local disease [5]. However, new studies have demonstrated that resection of solitary or limited metastatic disease, also called ‘oligometastases’ [6], may improve outcomes. There is evidence for favorable outcomes for surgical resection of oligometastatic lung cancer with isolated adrenal and brain metastasis [7][8]. We present an interesting case of surgical resection for oligometastatic metachronous lung cancer to the contralateral scapula in a patient who had previously undergone salvage pneumonectomy after radical chemo-radiotherapy.

## Case presentation

**Figure 1:**
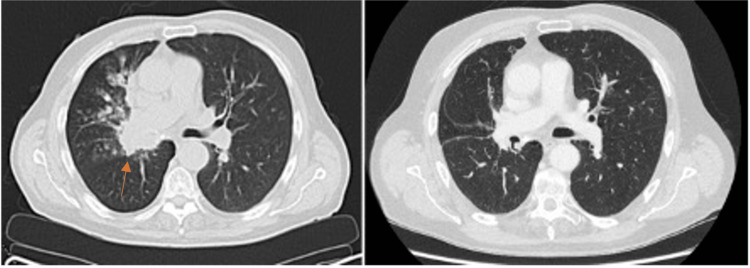
Initial CT scan demonstrating right perihilar lesion (left panel). CT scan post-chemo-radiotherapy demonstrating a reduction in tumor size (right panel).

A 74-year-old male, with a sixty-pack-year smoking history, was initially investigated for a persistent dry cough. Further imaging and biopsy led to the diagnosis of T4 N1 M0 squamous cell carcinoma of the right hilum (primary lesion encasing the right main bronchus, not causing collapse). He was treated with concurrent chemo-radiotherapy with curative intent, consisting of four cycles of Carboplatin and Vinorelbine, and radiotherapy in the form of 55Gy in 20 fractions. His oncological treatment was successfully completed a month later, with a good radiological response (Figure 1). 

**Figure 2:**
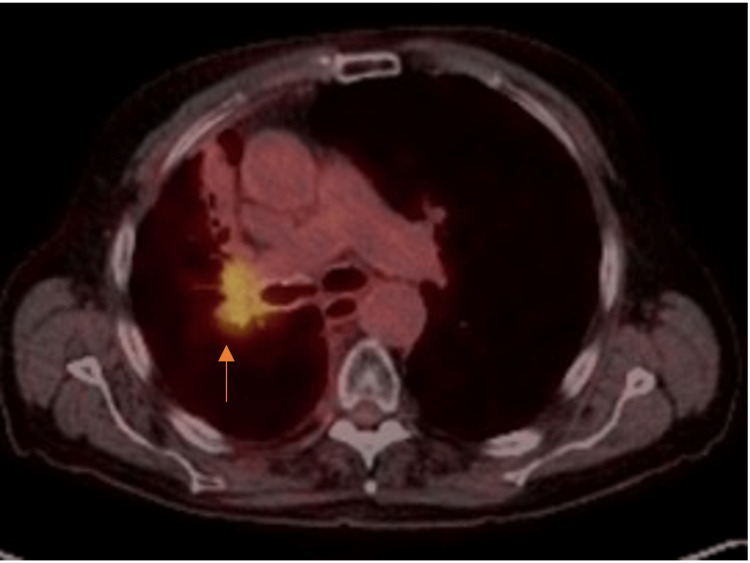
Follow-up PET scan demonstrating disease recurrence in the right perihilar region

Nine months later, follow-up imaging with a [18F]-fluorodeoxyglucose positron emission tomography/computed tomography (FDG-PET/CT) showed local recurrence (Figure 2).

Following multidisciplinary discussion, he underwent a right posterolateral thoracotomy and salvage pneumonectomy, recovering well post-operatively. Histology confirmed a fully resected ypT2a ypN0 squamous cell carcinoma (yp prefix indicating pathological staging post systemic treatment). The lesion measured thirty-one millimetres in maximum diameter. The postoperative course was uneventful.

**Figure 3:**
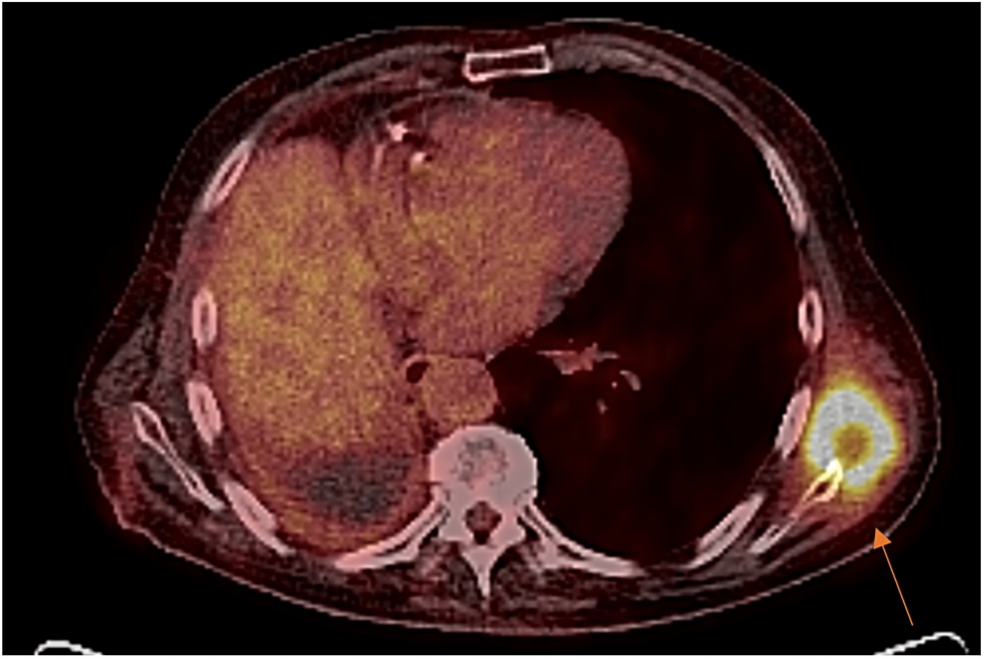
[18F]-fluorodeoxyglucose positron emission tomography/computed tomography (FDG-PET/CT) demonstrating left posterior chest wall metastasis

Seven months later, the patient experienced some left shoulder pain and subsequently a CT scan demonstrated a 52 x 48 x 42mm subcutaneous mass on the left posterior chest wall. Further imaging proved the lesion to be FDG-avid (figure 3) and a biopsy confirmed a squamous cell carcinoma metastasis isolated to the left chest wall adjacent to the scapula. 

No evidence of extension into the ribs or thoracic cavity was found. There was no evidence of further metastases. Following a multidisciplinary discussion, it was deemed that radiotherapy to the lesion would not be possible due to the risk of radiation-induced lung injury of the remaining lung. The metastasis appeared to be potentially resectable and the patient subsequently consented to left chest wall resection and reconstruction. The procedure involved a 22cm oblique incision over the chest wall mass. The latissimus dorsi was found uninvolved, and therefore, spared, and the mass was identified as arising from the angle of the scapula to the medial spine. A successful en block resection of the scapula and serratus anterior muscle was conducted with good margins. The thoracic cavity was not entered. Reconstruction of the scapula and serratus anterior was done using a 5x5cm 1.5mm thick porcine dermal collagen implant (PermacolTM Medtronic UK). A Redivac drain was sited, and this was followed by routine layered closure. 

In the immediate postoperative period, the patient developed a left-sided tension pneumothorax requiring urgent decompression with a surgical chest drain. This was performed successfully, following which he was transferred to the critical care unit for monitoring. Ultimately, he was discharged home upon resolution of these issues. Histology of the left chest mass confirmed lung squamous cell carcinoma with clear resection margins. At 6 months post-surgery, there continues to be no evidence of cancer recurrence. 

## Discussion

With advances in oncological management for patients with NSCLC, the number of patients who are now receiving treatment with curative intent is ever-increasing. Invariably there are cases where disease relapse occurs post-chemo-radiotherapy/immunotherapy. If such disease is confined to local spread, then a so-called ‘salvage surgery’ may be attempted. Salvage surgery is generally accepted to encompass surgical resection of local disease recurrence following the failure of prior treatments. However, it does not include surgical management of distant metastases [9]. In a study by Li et al., salvage surgery with lung resection following immunotherapy treatment failure in NSCLC was shown to be associated with improved progression-free survival compared with immunotherapy alone (23.4 months vs 12.9 months p< 0.0004) [10]. In addition, an analysis of 27 patients by Romero-Vielva et al. demonstrated that salvage surgery consisting of either lobectomy, bilobectomy, or pneumonectomy in NSCLC post definitive chemo-radiotherpay was associated with an overall survival (time between surgical resection and last follow-up) of 75 months [11]. 

Whilst local disease spread may benefit from ‘salvage surgery’, indications for surgery in distant metastatic NSCLC remain less clear. With the advent and widespread availability of highly sensitive imaging modalities, distant solitary or limited so-called ‘oligometastases’ are becoming increasingly identified posing challenges to clinicians in terms of identifying the optimum management strategy. At present, the use of stereotactic body radiotherapy (SBRT) is gaining popularity due to its non-invasive nature and relatively low side-effect burden. A randomized open-label phase 2 trial involving 99 patients has demonstrated that compared to standard palliative treatment, SBRT was associated with improved overall survival of 41 months vs 28 months in standard palliative treatment (hazard ratio 0.57 95% CI 0.3-1.1) [12]. 

Whilst there are no direct comparisons of radiotherapy and surgery in the management of oligometastases, there are studies demonstrating the potential benefit of surgical resection in certain cases. In a retrospective analysis of 37 patients with NSCLC who underwent surgical resection of isolated adrenal metastases, a survival benefit at 5 years was shown when compared to non-operative treatment (34% vs 0% p< 0.002) [13]. In addition, a 10-year multicentre retrospective study carried out by De Wolf et al. involving 59 patients with adrenalectomy in oligometastatic NSCLC was shown to be associated with a 5-year survival rate of 59% [14]. It should be noted that 40% of patients received additional treatment with chemo/radiotherapy post metastasectomy. In a study of 36 NSCLC patients with solitary brain metastases with mutations to epidermal growth factor receptor (eGFR), surgical resection of the brain and lung lesions followed by immunotherapy was shown to lead to a survival benefit when compared to radiotherapy followed by immunotherapy (28 vs 16 months p< 0.044) [15]. Data for oligometastases of the skeletal system is rare; however, a case report by Hirano et al. did demonstrate that resection of solitary skeletal metastases in patients with NSCLC oligometastases was associated with no disease recurrence at 5 years [16]. 

## Conclusions

This case report is unique in that it combines both salvage surgery and subsequent surgical management of skeletal oligometastases. Although isolated skeletal metastases post salvage pneumonectomy is likely to be a rare occurrence, due to the risk of radiation-associated lung injury, radiotherapy is not a viable option in this patient cohort. Surgical resection of oligometastatic disease was possible in this case as patient fitness was maintained following initial treatment and salvage surgery. However, in cases where fitness is not maintained, such an approach would be precluded. 

In this case, we have demonstrated that an isolated skeletal metastasis may be successfully managed with surgical resection and chest wall reconstruction. Whilst the patient developed a complication in the form of a tension pneumothorax in the immediate postoperative period, the long-term outcome was favorable. In such complex cases, a tailored approach involving a multidisciplinary team and careful consideration of the patient’s circumstances is necessary for a satisfactory outcome. Further studies are likely to be required to evaluate longer-term outcomes and feasibility in a larger patient population. 
